# Supplementary material for: Analysis of the Direct Medical Costs of Colorectal Cancer in Antigua and Barbuda: A Prevalence-Based Cost-of-Illness Study
Source: Int J Environ Res Public Health. 2025 Apr 3;22(4):552. doi: 10.3390/ijerph22040552 (PMC12027121; doi:10.3390/ijerph22040552)
Supplement: Supplementary file 1 [file ijerph-22-00552-s001.zip › Supplementary file 2.pdf]

Supplementary file 2:

Table showing the direct medical unit costs for staging, management and treatment of rectal cancer stages I-IV

**Staging and treatment variables**	**Unit Costs (USD)**	**Clinical Stage**			
**Estimated Number of Cases in a Single Year (N=4)**		**I (n=1)**	**II (n=1)**	**III (n=1)**	**IV (n=1)**
**Diagnosis and Imaging**					
Consultation (Clinical Assessment, Physical Examination)	$147.23	$147.23	$147.23	$147.23	$147.23
Guaiac-Fecal Occult Blood Test	$14.72	$14.72	$-	$-	$-
Colonoscopy	$1,288.23	$1,288.23	$1,288.23	$1,288.23	$1,288.23
Biopsy	$368.07	$368.07	$368.07	$368.07	$368.07
Imaging (Radiology)	$1,503.18	$1,503.18	$1,503.18	$1,503.18	$1,503.18
Laboratory	$530.02	$530.02	$530.02	$530.02	$530.02
Histopathology	$628.66	$628.66	$628.66	$628.66	$628.66
**Treatment (Average Colorectal Cancer Cases in a Single Year=11)**					
Surgery (Lymphadenectomy)	$7,315.10	$7,315.10	$7,315.10	$7,315.10	$-
Surgery (resection)	$15,889.32	$-	$-	$-	$15,889.32
Radiotherapy (External Beam Radiotherapy-EBRT)	$12,974.35	$-	$-	$12,974.35	$12,974.35
Systemic therapy (Chemotherapy)*	$7,926.31	$-	$7,926.31	$7,926.31	$7,926.31
Immunotherapy	$19,200.00	$-	$19,200.00	$19,200.00	$19,200.00
**Post-treatment side-effects care**					
Blood clot prophylaxis	$360.00	$360.00	$360.00	$360.00	$360.00
Renal complaints	$3,763.61	$-	$-	$3,763.61	$3,763.61

Anaemia (Low Hemoglobin/Hematocrit)	$6,687.76	$6,687.76	$6,687.76	$6,687.76	$6,687.76
Infections Control	$365.00	$290.42	$290.42	$290.42	$290.42
Other Complications of Treatment (post-operative pain, wound care, other issues etc.)	$28,469.72	$13,942.77	$13,942.77	$13,942.77	$13,942.77
**Other direct costs**					
Nutrition Counselling	$100.00	$100.00	$100.00	$100.00	$100.00
Psychiatric/psychological Counselling	$128.82	$128.82	$128.82	$128.82	$128.82
Pharmacy Services	$89.99	$89.99	$89.99	$89.99	$89.99
Positron Emission Tomography (PET) Scan (Overseas)**	$991.94	$991.94	$991.94	$991.94	$991.94
Chemotherapy Port Insertion	$7,361.33	$7,361.33	$7,361.33	$7,361.33	$7,361.33
Emergency Kit (Chemo)	$470.83	$470.83	$470.83	$470.83	$470.83
Patient Transportation/Accommodation (overseas imaging)**	$1,398.65	$1,398.65	$1,398.65	$1,398.65	$1,398.65
Transportation (local)	$561.30	$561.30	$561.30	$561.30	$561.30
Overheads	$36.81	$36.81	$36.81	$36.81	$36.81
**Ongoing Care**					
Follow-up Consultations	$368.07	$368.07	$368.07	$368.07	$368.07
Imaging Studies (CT scan, chest X-ray, echocardiogram)	$975.38	$975.38	$975.38	$975.38	$975.38
Biochemistry Tests (chemistry/renal panel, liver function tests, HbA1c, cholesterol)	$1509.09	$1509.09	$1509.09	$1509.09	$1509.09
**Total (crude estimates)**	**$119,032.90**	**$73,541.46**	**$100,653.05**	**$104,416.66**	**$81,212.24**
**Total (revised estimates)**	**$121,723.50**	**$76,232.05**	**$103,343.64**	**$107,107.25**	**$83,902.83**

*****Outsourced cancer care services linked to overseas care***

Crude estimates: the costs of outsourced cancer care services are exempt from the analysis

Revised estimates: the costs of outsourced cancer care services are included in the analysis
