# Supplementary material for: Analysis of the Direct Medical Costs of Colorectal Cancer in Antigua and Barbuda: A Prevalence-Based Cost-of-Illness Study
Source: Int J Environ Res Public Health. 2025 Apr 3;22(4):552. doi: 10.3390/ijerph22040552 (PMC12027121; doi:10.3390/ijerph22040552)
Supplement: Supplementary file 1 [file ijerph-22-00552-s001.zip › Supplementary file 3.pdf]

**Supplementary file 3**

**Table showing the total annual costs estimation for colon cancer (direct medical costs) (estimated cases=10)**

| Parameter | Care Component/Procedures | Average Number of Cases in a Single Year (N=10) | Estimated Average Cost 2021 (USD) | Total Costs (USD) | Sum-total & Percentage of Cost (adjusted) | Range (USD)± 25% | |
|---|---|---|---|---|---|---|---|
| | | | | | | Lower | Upper |
| **Diagnosis and Imaging** | **Diagnosis and Imaging** | | | | | | |
| | Consultation (Clinical assessment/Physical examination) | 10 | $147.23 | $1,472.30 | | $1,104.23 | $1,840.38 |
| | Guaiac-Fecal Occult Blood Test | 10 | $14.72 | $147.20 | | $110.40 | $184.00 |
| | Colonoscopy | 10 | $1,288.23 | $12,882.30 | | $9,661.73 | $16,102.88 |
| | Biopsy | 10 | $368.07 | $3,680.70 | | $2,760.53 | $4,600.88 |
| | Imaging (Radiology) | 10 | $1,503.18 | $15,031.80 | | $11,273.85 | $18,789.75 |
| | Laboratory | 10 | $530.02 | $5,300.20 | | $3,975.15 | $6,625.25 |
| | Histopathology | 10 | $628.66 | $6,286.60 | | $4,714.95 | $7,858.25 |
| *Subtotal* | | | | ***$44,801.10*** | ***4.43%*** | ***$33,600.83*** | ***$56,001.38*** |
| **Treatment** | **Treatment** | | | | | | |
| | Stage I | 2 | $27,577.78 | $55,155.56 | | $41,366.67 | $68,944.45 |
| | Stage II | 3 | $54,704.09 | $164,112.27 | | $123,084.20 | $205,140.34 |
| | Stage III | 4 | $67,678.44 | $270,713.76 | | $203,035.32 | $338,392.20 |
| | Stage IV | 1 | $40,100.66 | $40,100.66 | | $30,075.50 | $50,125.83 |
| *Subtotal* | | | | ***$530,082.25*** | ***52.46%*** | ***$397,561.69*** | ***$662,602.81*** |
| **Post-treatment care** | **Post-treatment care** | | | | | | |
| | Blood clot prophylaxis | 10 | $360.00 | $3,600.00 | | $2,700.00 | $4,500.00 |
| | Renal complaint | 2 | $3,763.61 | $7,527.22 | | $5,645.42 | $9,409.03 |

| | | | | | | | |
|---|---|---|---|---|---|---|---|
| | Anaemia (low Hemoglobin/Hematocrit) | 10 | $6,687.76 | $66,877.60 | | $50,158.20 | $83,597.00 |
| | Infections Control | 10 | $365.00 | $3,650.00 | | $2,737.50 | $4,562.50 |
| | Other Complications of Treatment | 10 | $28,469.72 | $284,697.20 | | $213,522.90 | $355,871.50 |
| *Subtotal* | | | | ***$366,352.02*** | ***36.25%*** | ***$274,764.02*** | ***$457,940.03*** |
| **Other Direct Medical Costs** | **Other direct costs** | | | | | | |
| | Nutrition Counselling | 10 | $100.00 | $1,000.00 | | $750.00 | $1,250.00 |
| | Psychiatric/psychological Counselling | 10 | $128.82 | $1,288.20 | | $966.15 | $1,610.25 |
| | Pharmacy Services | 10 | $89.99 | $899.90 | | $674.93 | $1,124.88 |
| | Positron Emission Tomography (PET) Scan (Overseas) | 2 | $991.94 | $1,983.88 | | $1,487.91 | $2,479.85 |
| | Chemotherapy Port Insertion | 3 | $7,361.33 | $22,083.99 | | $16,562.99 | $27,604.99 |
| | Emergency Kit (Chemo) | 10 | $470.83 | $4,708.30 | | $3,531.23 | $5,885.38 |
| | Patient Transportation/Accommodation (overseas imaging) | 2 | $1,398.65 | $2,797.30 | | $2,097.98 | $3,496.63 |
| | Transportation (local) | 10 | $561.30 | $5,613.00 | | $4,209.75 | $7,016.25 |
| | Overheads | 10 | $36.81 | $368.10 | | $276.08 | $460.13 |
| *Subtotal* | | | | ***$40,742.67*** | ***4.03%*** | ***$30,557.00*** | ***$50,928.34*** |
| **Ongoing Care** | **Ongoing Care** | | | | | | |
| | Follow-up Consultations | 10 | $368.07 | $3,680.70 | | $2,760.53 | $4,600.88 |
| | Imaging Studies (CT scan, chest X-ray, echocardiogram) | 10 | $975.38 | $9,753.80 | | $7,315.35 | $12,192.25 |
| | Biochemistry Tests (chemistry/renal panel, liver | 10 | $1,509.09 | $15,090.90 | | $11,318.18 | $18,863.63 |

| | | | | | | | |
|---|---|---|---|---|---|---|---|
| *Subtotal* | function tests, HbA1c, cholesterol) | | | ***$28,525.40*** | ***2.82%*** | ***$21,394.05*** | ***$35,656.75*** |
| **Total Direct Medical Costs (crude estimates)** | | | | **$1,005,722.26** | | **$754,291.70** | **$1,257,152.83** |
| **Total Direct Medical Costs (revised estimates)** | | | | **$1,010,503.44** | | **$757,877.58** | **$1,263,129.30** |

***Outsourced cancer care services linked to overseas care*

***Crude estimates: the costs of outsourced cancer care services are exempt from the analysis***

***Revised estimates: the costs of outsourced cancer care services are included in the analysis***
