# Supplementary material for: Analysis of the Direct Medical Costs of Colorectal Cancer in Antigua and Barbuda: A Prevalence-Based Cost-of-Illness Study
Source: Int J Environ Res Public Health. 2025 Apr 3;22(4):552. doi: 10.3390/ijerph22040552 (PMC12027121; doi:10.3390/ijerph22040552)
Supplement: Supplementary file 1 [file ijerph-22-00552-s001.zip › Supplementary file 4.pdf]

Supplementary file 4

Table showing the total annual costs estimation for rectal cancer (direct medical costs) (estimated cases=4)

Parameter	Care Component/Procedures	Average Number of Cases in a Single Year (N=4)	Estimated Average Cost 2021 (USD)	Total Costs (USD)	Sum-total & Percentage of Cost (adjusted)	Range (USD)± 25%	
						Lower	Upper
**Diagnosis and Imaging**	**Diagnosis and Imaging**						
	Consultation (Clinical assessment/Physical examination)	4	$147.23	$588.92		$441.69	$736.15
	Guaiac-Fecal Occult Blood Test	4	$14.72	$58.88		$44.16	$73.60
	Colonoscopy	4	$1,288.23	$5,152.92		$3,864.69	$6,441.15
	Biopsy	4	$368.07	$1,472.28		$1,104.21	$1,840.35
	Imaging (Radiology)	4	$1,503.18	$6,012.72		$4,509.54	$7,515.90
	Laboratory	4	$530.02	$2,120.08		$1,590.06	$2,650.10
	Histopathology	4	$628.66	$2,514.64		$1,885.98	$3,143.30
Subtotal				***$17,920.44***	***4.54%***	***$13,440.33***	***$22,400.55***
**Treatment**	**Treatment**						
	Stage I	1	$36,178.77	$36,178.77		$27,134.08	$45,223.46
	Stage II	1	$63,305.08	$63,305.08		$47,478.81	$79,131.35
	Stage III	1	$63,305.08	$63,305.08		$47,478.81	$79,131.35
	Stage IV	1	$40,100.66	$40,100.66		$30,075.50	$50,125.83
Subtotal				***$202,889.59***	***51.39%***	***$152,167.19***	***$253,611.99***
**Post-treatment care**	**Post-treatment care**						
	Blood clot prophylaxis	4	$360.00	$1,440.00		$1,080.00	$1,800.00
	Renal complaint	1	$3,763.61	$3,763.61		$2,822.71	$4,704.51

	Anaemia (low Hemoglobin/Hematocrit)	4	$6,687.76	$26,751.04		$20,063.28	$33,438.80
	Infections Control	4	$365.00	$1,460.00		$1,095.00	$1,825.00
	Other Complications of Treatment	4	$28,469.72	$113,878.88		$85,409.16	$142,348.60
Subtotal				***$147,293.53***	***37.31%***	***$110,470.15***	***$184,116.91***
**Other Direct Medical Costs**	**Other direct costs**						
	Nutrition Counselling	4	$100.00	$400.00		$300.00	$500.00
	Psychiatric/psychological Counselling	4	$128.82	$515.28		$386.46	$644.10
	Pharmacy Services	4	$89.99	$359.96		$269.97	$449.95
	Positron Emission Tomography (PET) Scan (Overseas)	1	$991.94	$991.94		$743.96	$1,239.93
	**Chemotherapy Port Insertion**	**1**	**$7,361.33**	$7,361.33		$5,521.00	$9,201.66
	Emergency Kit (Chemo)	4	$470.83	$1,883.32		$1,412.49	$2,354.15
	Patient Transportation/Accommodation (overseas imaging)	1	$1,398.65	$1,398.65		$1,048.99	$1,748.31
	Transportation (local)	4	$561.30	$2,245.20		$1,683.90	$2,806.50
	Overheads	4	$36.81	$147.24		$110.43	$184.05
Subtotal				***$15,302.92***	***3.88%***	***$11,477.19***	***$19,128.65***
**Ongoing Care**	**Ongoing Care**						
	Follow-up Consultations	4	$368.07	$1,472.28		$1,104.21	$1,840.35
	Imaging Studies (CT scan, chest X-ray, echocardiogram)	4	$975.38	$3,901.52		$2,926.14	$4,876.90
	Biochemistry Tests (chemistry/renal panel, liver	4	$1509.09	$6,036.36		$4,527.27	$7,545.45

Subtotal	function tests, HbA1c, cholesterol)			***$11,410.16***	***2.89%***	***$8,557.62***	***$14,262.70***
**Total Direct Medical Costs (crude estimates)**				**$392,426.05**		**$294,319.54**	**$490,532.56**
**Total Direct Medical Costs (revised estimates)**				**$394,816.64**		**$296,112.48**	**$493,520.80**

*****Outsourced cancer care services linked to overseas care***

Crude estimates: the costs of outsourced cancer care services are exempt from the analysis

Revised estimates: the costs of outsourced cancer care services are included in the analysis
