# Supplementary material for: Analysis of the Direct Medical Costs of Colorectal Cancer in Antigua and Barbuda: A Prevalence-Based Cost-of-Illness Study
Source: Int J Environ Res Public Health. 2025 Apr 3;22(4):552. doi: 10.3390/ijerph22040552 (PMC12027121; doi:10.3390/ijerph22040552)
Supplement: Supplementary file 1 [file ijerph-22-00552-s001.zip › Supplementary file 5.pdf]

Supplementary file 5

Table showing total annual costs estimation for colon cancer (direct medical costs) (estimated cases=15) (50% increase in average prevalence)

**Parameter**	**Care Component/Procedures**	**Average Number of Cases in a Single Year (N=15)**	**Estimated Average Cost 2021 (USD)**	**Total Costs (USD)**	**Sum-total & Percentage of Cost (adjusted)**	**Range (USD)± 25%**	
						**Lower**	**Upper**
**Diagnosis and Imaging**	**Diagnosis and Imaging**						
	Consultation (Clinical assessment/Physical examination)	15	$147.23	$2,208.45		$1,656.34	$2,760.56
	Guaiac-Fecal Occult Blood Test	15	$14.72	$220.80		$165.60	$276.00
	Colonoscopy	15	$1,288.23	$19,323.45		$14,492.59	$24,154.31
	Biopsy	15	$368.07	$5,521.05		$4,140.79	$6,901.31
	Imaging (Radiology)	15	$1,503.18	$22,547.70		$16,910.78	$28,184.63
	Laboratory	15	$530.02	$7,950.30		$5,962.73	$9,937.88
	Histopathology	15	$628.66	$9,429.90		$7,072.43	$11,787.38
Subtotal				***$67,201.65***	***4.48%***	***$50,401.24***	***$84,002.06***
**Treatment**	**Treatment**						
	Stage I	3	$27,577.78	$82,733.34		$62,050.01	$103,416.68
	Stage II	4	$54,704.09	$218,816.36		$164,112.27	$273,520.45
	Stage III	6	$67,678.44	$406,070.64		$304,552.98	$507,588.30
	Stage IV	2	$40,100.66	$80,201.32		$60,150.99	$100,251.65
Subtotal				***$787,821.66***	***52.57%***	***$590,866.25***	***$984,777.08***
**Post-treatment care**	**Post-treatment care**						
	Blood clot prophylaxis	15	$360.00	$5,400.00		$4,050.00	$6,750.00
	Renal complaint	2	$3,763.61	$7,527.22		$5,645.42	$9,409.03

	Anaemia (low Hemoglobin/Hematocrit)	15	$6,687.76	$100,316.40		$75,237.30	$125,395.50
	Infections Control	15	$365.00	$5,475.00		$4,106.25	$6,843.75
	Other Complications of Treatment	15	$28,469.72	$427,045.80		$320,284.35	$533,807.25
Subtotal				***$545,764.42***	***36.42%***	***$409,323.32***	***$682,205.53***
**Other Direct Medical Costs**	**Other direct costs**						
	Nutrition Counselling	15	$100.00	$1,500.00		$1,125.00	$1,875.00
	Psychiatric/psychological Counselling	15	$128.82	$1,932.30		$1,449.23	$2,415.38
	Pharmacy Services	15	$89.99	$1,349.85		$1,012.39	$1,687.31
	Positron Emission Tomography (PET) Scan (Overseas)	2	$991.94	$1,983.88		$1,487.91	$2,479.85
	Chemotherapy Port Insertion	4	$7,361.33	$29,445.32		$22,083.99	$36,806.65
	Emergency Kit (Chemo)	15	$470.83	$7,062.45		$5,296.84	$8,828.06
	Patient Transportation/Accommodation (overseas imaging)	2	$1,398.65	$2,797.30		$2,097.98	$3,496.63
	Transportation (local)	15	$561.30	$8,419.50		$6,314.63	$10,524.38
	Overheads	15	$36.81	$552.15		$414.11	$690.19
Subtotal				***$55,042.75***	***3.67%***	***$41,282.06***	***$68,803.44***
**Ongoing Care**	**Ongoing Care**						
	Follow-up Consultations	15	$368.07	$5,521.05		$4,140.79	$6,901.31
	Imaging Studies (CT scan, Chest X-ray, Echocardiogram)	15	$975.38	$14,630.70		$10,973.03	$18,288.38
	Biochemistry Tests (chemistry/renal panel, liver	15	$1,509.09	$22,636.35		$16,977.26	$28,295.44

Subtotal	function tests, HbA1c, cholesterol)			***$42,788.10***	***2.86%***	***$32,091.08***	***$53,485.13***
**Total Direct Medical Costs (crude estimates)**				**$1,493,837.40**		**$1,120,378.05**	**$1,867,296.75**
**Total Direct Medical Costs (revised estimates)-C**				**$1,498,618.58**		**$1,123,963.94**	**$1,873,273.23**

***Outsourced cancer care services linked to overseas care*

Crude estimates: the costs of outsourced cancer care services are exempt from the analysis

Revised estimates: the costs of outsourced cancer care services are included in the analysis
