# Supplementary material for: Analysis of the Direct Medical Costs of Colorectal Cancer in Antigua and Barbuda: A Prevalence-Based Cost-of-Illness Study
Source: Int J Environ Res Public Health. 2025 Apr 3;22(4):552. doi: 10.3390/ijerph22040552 (PMC12027121; doi:10.3390/ijerph22040552)
Supplement: Supplementary file 1 [file ijerph-22-00552-s001.zip › Supplementary file 6.pdf]

Supplementary file 6

Table showing total annual costs estimation for rectal cancer (direct medical costs) (estimated cases=6) (50% increase in average prevalence)

**Parameter**	**Care Component/Procedures**	**Average Number of Cases in a Single Year (N=6)**	**Estimated Average Cost 2021 (USD)**	**Total Costs (USD)**	**Sum-total & Percentage of Cost (adjusted)**	**Range (USD)± 25%**	
						**Lower**	**Upper**
**Diagnosis and Imaging**	**Diagnosis and Imaging**						
	Consultation (Clinical assessment/Physical examination)	6	$147.23	$883.38		$662.54	$1,104.23
	Guaiac-Fecal Occult Blood Test	6	$14.72	$88.32		$66.24	$110.40
	Colonoscopy	6	$1,288.23	$7,729.38		$5,797.04	$9,661.73
	Biopsy	6	$368.07	$2,208.42		$1,656.32	$2,760.53
	Imaging (Radiology)	6	$1,503.18	$9,019.08		$6,764.31	$11,273.85
	Laboratory	6	$530.02	$3,180.12		$2,385.09	$3,975.15
	Histopathology	6	$628.66	$3,771.96		$2,828.97	$4,714.95
Subtotal				***$26,880.66***	***4.52%***	***$20,160.50***	***$33,600.83***
**Treatment**	**Treatment**						
	Stage I	1	$36,178.77	$36,178.77		$27,134.08	$45,223.46
	Stage II	1	$63,305.08	$63,305.08		$47,478.81	$79,131.35
	Stage III	2	$63,305.08	$126,610.16		$94,957.62	$158,262.70
	Stage IV	2	$40,100.66	$80,201.32		$60,150.99	$100,251.65
Subtotal				***$306,295.33***	***51.50%***	***$229,721.50***	***$382,869.16***
**Post-treatment care**	**Post-treatment care**						
	Blood clot prophylaxis	6	$360.00	$2,160.00		$1,620.00	$2,700.00
	Renal complaint	1	$3,763.61	$3,763.61		$2,822.71	$4,704.51

	Anaemia (low Hemoglobin/Hematocrit)	6	$6,687.76	$40,126.56		$30,094.92	$50,158.20
	Infections Control	6	$365.00	$2,190.00		$1,642.50	$2,737.50
	Other Complications of Treatment	6	$28,469.72	$170,818.32		$128,113.74	$213,522.90
Subtotal				***$219,058.49***	***36.83%***	***$164,293.87***	***$273,823.11***
**Other Direct Medical Costs**	**Other direct costs**						
	Nutrition Counselling	6	$100.00	$600.00		$450.00	$750.00
	Psychiatric/psychological Counselling	6	$128.82	$772.92		$579.69	$966.15
	Pharmacy Services	6	$89.99	$539.94		$404.96	$674.93
	Positron Emission Tomography (PET) Scan (Overseas)	1	$991.94	$991.94		$743.96	$1,239.93
	Chemotherapy Port Insertion	2	$7,361.33	$14,722.66		$11,042.00	$18,403.33
	Emergency Kit (Chemo)	6	$470.83	$2,824.98		$2,118.74	$3,531.23
	Patient Transportation/Accommodation (overseas imaging)	1	$1,398.65	$1,398.65		$1,048.99	$1,748.31
	Transportation (local)	6	$561.30	$3,367.80		$2,525.85	$4,209.75
	Overheads	6	$36.81	$220.86		$165.65	$276.08
Subtotal				***$25,439.75***	***4.28%***	***$19,079.81***	***$31,799.69***
**Ongoing Care**	**Ongoing Care**						
	Follow-up Consultations	6	$368.07	$2,208.42		$1,656.32	$2,760.53
	Imaging Studies (CT scan, Chest X-ray, Echocardiogram)	6	$975.38	$5,852.28		$4,389.21	$7,315.35
	Biochemistry tests (chemistry/renal panel, liver	6	$1,509.09	$9,054.54		$6,790.91	$11,318.18

	function tests, HbA1c, cholesterol)						
Subtotal				***$17,115.24***	***2.88%***	***$12,836.43***	***$21,394.05***
**Total Direct Medical Costs (crude estimates)**				**$592,398.88**		**$444,299.16**	**$740,498.60**
**Total Direct Medical Costs (revised estimates)-R**				**$594,789.47**		**$446,092.10**	**$743,486.84**

*****Outsourced cancer care services linked to overseas care***

Crude estimates: the costs of outsourced cancer care services are exempt from the analysis

Revised estimates: the costs of outsourced cancer care services are included in the analysis
